# ESPO AND HEALTH SCIENCES SECTION SYMPOSIUM: INCLUDING DIVERSE STAKEHOLDERS IN HEALTH INTERVENTION DESIGN: LESSONS LEARNED FOR ADVANCING HEALTH EQUITY

**DOI:** 10.1093/geroni/igac059.1035

**Published:** 2022-12-20

**Authors:** An Nguyen, Kyle Moored, Kyle Moored

## Abstract

The COVID-19 pandemic exposed persistent disparities in the effectiveness of healthcare services and long-term care among the older adult population. For example, significant gaps disproportionately affected older adults living in rural areas, racial and ethnic minorities, individuals with cognitive impairments, and those from low socioeconomic backgrounds. To improve health equity, research is needed to design, implement, and disseminate health interventions that address the heterogeneity of older adults and caregivers. Reflecting on lessons learned from including underrepresented key stakeholder groups in intervention research may reveal strategies to design health interventions that are more accessible and effective for diverse populations of older adults and their caregivers. This symposium highlights five studies that included diverse populations of older adults and/or caregivers in the design of health interventions. Dr. Schiaffino will share how disparities in co-morbid cancer and dementia across racial, ethnic, and age groups will inform improvements to care delivery processes for this population. Ms. Crane will discuss barriers to designing exergame interventions that are accessible to older adults with multiple chronic conditions and functional limitations. Dr. Díaz-Santos will describe results of piloting and refining a social connection intervention to reduce loneliness among Latinx older adults at risk for dementia. Dr. del Pino will highlight strategies to improve implementation of a screening intervention for at-risk drinking among African American older adults living with HIV. Mr. Cotton will present results of tailoring a crisis intervention to improve cultural fit for African American caregivers of people with dementia.

